# Prevalence of *Borrelia*, *Neoehrlichia mikurensis* and *Babesia* in ticks collected from vegetation in eastern Poland

**DOI:** 10.1007/s10493-023-00818-y

**Published:** 2023-06-30

**Authors:** Anna Sawczyn-Domańska, Jacek Zwoliński, Anna Kloc, Angelina Wójcik-Fatla

**Affiliations:** Department of Health Biohazards and Parasitology, Institute of Rural Health, Jaczewskiego 2, 20-090 Lublin, Poland

**Keywords:** *Borrelia burgdorferi* sensu lato, *Borrelia miyamotoi*, *Neoehrlichia mikurensis*, *Babesia* spp., *Ixodes ricinus*, *Dermacentor reticulatus*

## Abstract

In Poland, tick-borne diseases constitute the majority of diseases related to exposure to biological agents with a predominance of Lyme borreliosis; therefore, research on ticks as a reservoir of various pathogens remains crucial in the epidemiology of human diseases after tick bites. This study aimed to identify the occurrence of *Borrelia burgdorferi* sensu lato, *Borrelia miyamotoi*, *Neoehrlichia mikurensis*, and *Babesia* spp. in ticks collected from vegetation in eastern Poland. Additionally, the prevalence of co-infections in the adult *Ixodes ricinus* ticks was determined. Among *I. ricinus* ticks the predominantly detected pathogen was *B. burgdorferi* s.l. (23%) with *B. burgdorferi* sensu stricto as the most frequently identified species, followed by *B. garinii*. In 2013, the double or triple infections of *B. burgdorferi* s.s., *B. afzelii*, and *B. garinii* species did not exceed 9% in adult ticks, whereas in 2016, the prevalence of mixed infections reached 29%. The prevalence of *N. mikurensis* and *B. miyamotoi* in *I. ricinus* was determined at the same level of 2.8%. Four *Babesia* species were identified in the examined *I. ricinus* population: *B. microti* (1.5%), *B. venatorum* (1.2%), *B. divergens* (0.2%), and *B. capreoli* (0.1%). Co-infections were detected in 10.1% of all infected ticks with the highest prevalence of co-infections with *B. burgdorferi* s.l. and *Babesia* species. The changes in the prevalence and the distribution of particular pathogens within tick populations indicate the need for monitoring the current situation related to tick-borne pathogens from the aspect of risk to human health.

**Supplementary Information:**

The online version contains supplementary material available at 10.1007/s10493-023-00818-y.

## Introduction

In Europe, ticks are among the most significant vectors and reservoirs of human and animal pathogens such as bacteria, protozoa, and viruses. The most prevalent tick-borne disease with excessive public health importance is Lyme borreliosis. The pathogens associated with Lyme borreliosis belong to the *Borrelia burgdorferi* sensu lato (s.l.) complex, which consists of 20 confirmed and three proposed species (Wolcott et al. 2021). In Europe, the human illness can be caused by at least five species (*B. garinii*, *B. afzelii*, *B. burgdorferi* sensu stricto [s.s.], *B. spielmanii*, *B. bavariensis*), of which most infections are caused by three: *B. garinii*, *B. afzelii*, and *B. burgdorferi* s.s. (Stanek et al. 2012; Steere et al. 2016). In Poland, Lyme borreliosis is the predominant human tick-borne disease in terms of disease incidence, namely, in 2010, the national incidence rate was 23.6 per 100,000 inhabitants, whereas in 2019, it reached 53.7 per 100,000 inhabitants (National Institute of Public Health-National Institute of Hygiene 2021).

*Borrelia miyamotoi*, a member of the relapsing fever group spirochaetes, was isolated for the first time from *I. persulcatus* in Japan in 1995 (Fukunaga et al. 1995). *Borrelia miyamotoi* and *B. burgdorferi* s.l. overlap in the geographic distribution and share the same tick vector species (Scoles et al. 2001; Fraenkel et al. 2002). In most cases, the symptoms of *B. miyamotoi* disease are mild and non-specific (including headache, fever, fatigue, muscle and joint pain) (Hoornstra et al. 2018; Franck et al. 2020), but in some patients, spirochetes can invade the central nervous system (Hovius et al. 2013; Henningsson et al. 2019).

*Neoehrlichia mikurensis* is a Gram-negative intracellular bacterium described for the first time in 2004 by Kawahara et al. (2004). Since then, *N. mikurensis* occurrence in *Ixodes* ticks has been demonstrated widely, both in Europe and Asia (Portillo et al. 2018). The first evidence of *N. mikurensis* pathogenicity for humans was reported in 2010 (Welinder-Olsson et al. 2010). Clinical manifestations of neoehrlichiosis vary from mild flu-like symptoms (fatigue, fever, myalgia, arthralgias) to severe illness with systemic inflammation (Pekova et al. 2011; Maurer et al. 2013).

Concerning protozoa, members of the genus *Babesia* comprise haemoprotozoan parasites transmitted by hard ticks, or less often via blood transfusion. Among > 100 identified *Babesia* species, four—*B. microti*, *B. divergens*, *B. duncani* and *B. venatorum*—have been confirmed as pathogenic to humans (Ngo and Civen 2009; Schnittger et al. 2012). Human babesiosis is an emerging infectious disease that in severe cases may lead to coma, renal failure, respiratory failure, and irregular heartbeat (Mørch et al. 2015).

Little is known about the distribution and frequency of occurrence of *N. mikurensis* and *B. miyamotoi* in Poland. Only a few studies have reported the presence of those pathogens in *I. ricinus* ticks in the country (Kiewra et al. 2014; Welc-Falęciak et al. 2014; Kowalec et al. 2017, 2019; Kubiak et al. 2019). Moreover, the role and importance of *Dermacentor reticulatus* ticks in the transmission of *N. mikurensis* and *B. miyamotoi* remain under investigation. Although *D. reticulatus* bites people much less frequently than *I. ricinus* (Lledó et al. 2014), they play a significant role as reservoirs for many pathogens of medical and veterinary importance, enabling their maintenance in the environment (Földvári et al. 2016). In previous studies we investigated the occurrence of *B. burgdorferi* s.l. and *Babesia* in *D. reticulatus* ticks from eastern Poland (Wójcik-Fatla et al. 2012, 2015; Zając et al. 2017), but there is lack of information about the occurrence of *N. mikurensis* and *B. miyamotoi* in the area. Owing to the increasing number of incidences of Lyme borreliosis, it is essential to conduct research to assess the risk of exposure to *B. burgdorferi* s.l., as well as to other tick-borne pathogens. Therefore, the presented study aimed to examine (1) the prevalence of *B. miyamotoi* and *N. mikurensis* in questing *I. ricinus* and *D. reticulatus* ticks from eastern Poland, the endemic area both for tick species and *B. burgdorferi* s.l., (2) the occurrence of *B. burgdorferi* s.l. and *Babesia* spp. in *I. ricinus*, and (3) the prevalence of co-infections in *I. ricinus* ticks.

## Materials and methods

### Tick collection and DNA extraction

**Fig. 1:**
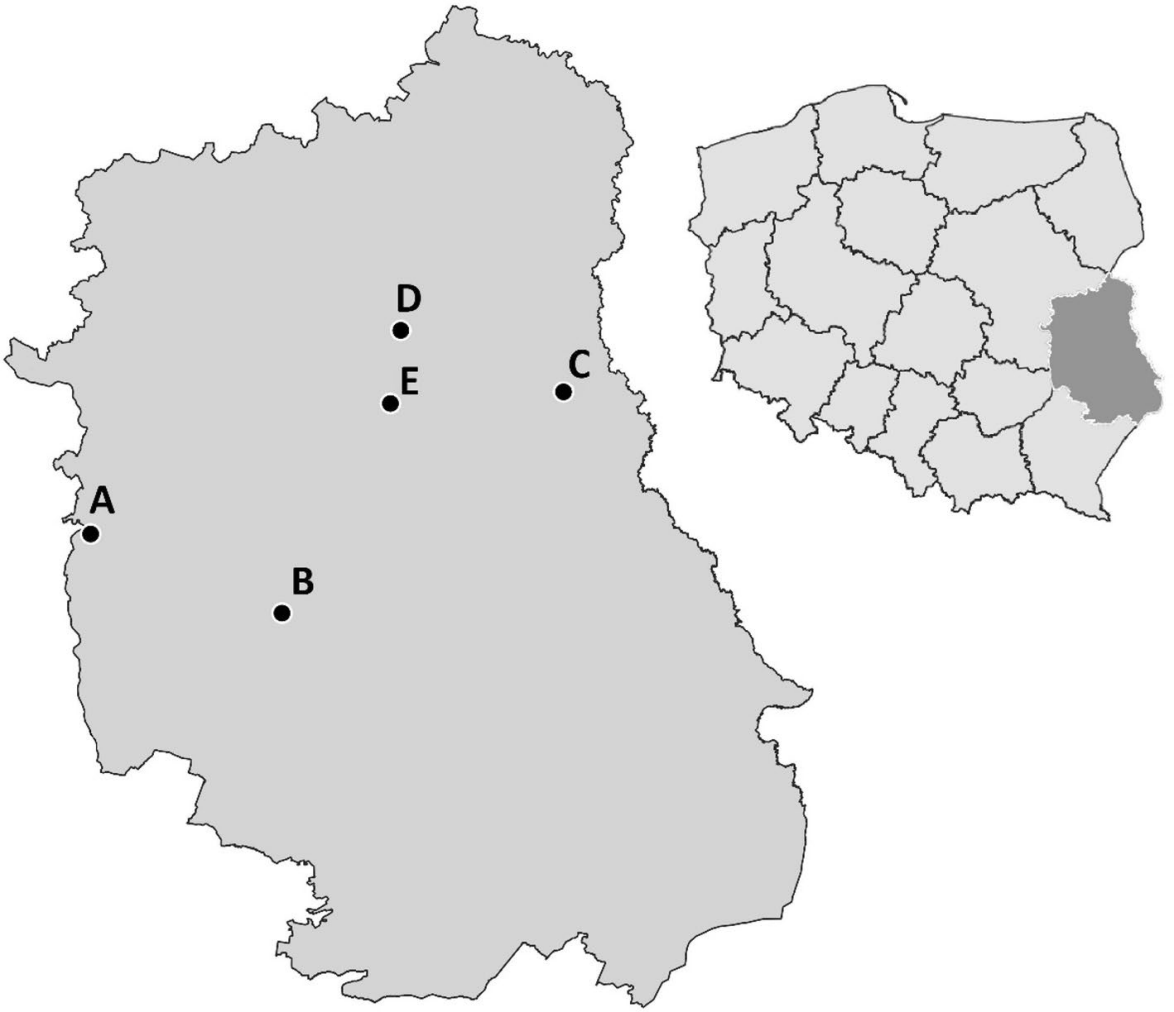
Geographical locations of five study areas in eastern Poland: Wilków (**A**), Piotrowice (**B**), Suchawa (**C**), Parczew (**D**), and Ostrów Lubelski (**E**). Map created in QGIS Software v.3.18.1-Zürich (QGIS.org 2021)

Questing ticks were collected from vegetation by the flagging method from 2010 to 2016, during the spring and autumn peak of the tick activity, in five natural and recreational locations in the Lublin Province of eastern Poland: Wilków (locality A), Piotrowice (B), Suchawa (C), Parczew (D), and Ostrów Lubelski (E) (Fig. 1). Total DNA from ticks was extracted by boiling in 0.7 M ammonium hydroxide (Rijpkema et al. 1996). Nymphs from localities B and C were pooled into groups containing five individuals, whereas nymphs from locality A and all adult ticks (males and females) were examined individually. DNA extracts were stored at − 20 °C for further study.

### Molecular identification of ticks

To confirm morphological identification of collected ticks, at least 10 specimens from each locality were intended for molecular research by polymerase chain reaction (PCR), based on the fragment of the mitochondrial 16 S rRNA gene (rDNA) with cycling conditions according to Black and Piesman (1994). The PCR assay was carried out in a 50 µl reaction solution containing 1 U of the Taq DNA Polymerase and reaction buffer with 15 mM MgCl_2_ (Qiagen, Germany), 0.5 µM of each primer (16S1 and 16S2), 0.2 mM of each dNTP, 1.5 µl DNA template and nuclease-free water.

### PCR detection of tick-borne pathogens

Detection of *B. burgdorferi* s.l. and identification of three *B. burgdorferi* species (*B. burgdorferi* s.s., *B. afzelii*, *B. garinii*) in *I. ricinus* ticks was carried out using PCR and a nested-PCR method that targeted a fragment of the *flaB* gene, according to the method described previously (Wójcik-Fatla et al. 2016). The positive control was DNA from the culture of *B. burgdorferi* s.l. isolated from *I. ricinus* ticks on the modified Barbour-Stoenner-Kelly (BSKH) medium (Cisak et al. 2006).

All ticks were screened for *Borrelia* belonging to the relapsing fever group using nested-PCR which amplified a fragment of the *p66* gene. All *p66*-positive DNA samples were then examined for the presence of *B. miyamotoi* by amplifying the *gplQ* gene, specific for *B. miyamotoi*. All PCR reactions were performed according to the procedure and cycling conditions described previously (Geller et al. 2012), with some modification. The volume reaction of 25 µl contained 0.625 U Taq DNA polymerase and buffer with 15 mM MgCl_2_ (Qiagen), 0.2 mM of each dNTP, 0.5 µM of each primer, 2 µl of DNA template for the first reaction, or 1 µl of the amplification product in the second reaction and nuclease-free water. The positive controls were isolates from *I. ricinus* ticks where *p66*/*glpQ* genes were detected and confirmed by sequencing.

For *N. mikurensis* detection, nested-PCR amplifying the 488-bp 16 S rRNA gene fragment was performed, based on the method by Richter and Matuschka (2012) with modification. All PCR reactions were performed in a final volume of 25 µl, containing: 1 U BIOTOOLS polymerase and buffer with 20 mM MgCl_2_ (Biotools, Spain), 0.2 mM of each dNTP, 0.5 µM of each primer, 1 µl DNA template (or 0.2 µl of the PCR product from the first reaction as a template for nested-PCR), and nuclease-free water. As a positive control, samples positive for *N. mikurensis* confirmed by sequencing were used.

DNA extracts were examined for *Babesia* spp. (including *B. divergens*, *B. venatorum*, *B. bigemina*, *B. major*, *B. canis*, *B. odocoilei*, *B. ovata*, *B. motasi*, *B. crassa*) in *I. ricinus* (from localities B and C) by amplification of the 18 S rRNA gene according to methods described previously (Hilpertshauser et al. 2006). Tick lysates confirmed by sequencing as positive for *B. divergens* and *B. venatorum* were used as a positive control. *Babesia microti* was detected using primers targeting the gene fragment encoding the nuclear small-subunit ribosomal RNA (SS-rDNA), according to the method described previously by Persing et al. (1992), with the modification described by Wójcik-Fatla et al. (2012). As a positive control, we used DNA isolated from a *B. microti*-infected hamster or mouse erythrocytes fixed on diagnostic slides (Fuller Laboratories, Germany).

Nuclease-free water was used as a negative control in all types of reactions. The primers used in this study are listed in supplementary material (Table S1). The PCR reactions were performed using C1000 Thermal Cycler (BioRad, USA) and Mastercycler Nexus (Eppendorf, Germany).

### DNA sequencing and sequence analysis

Sequencing of PCR products was performed with the ABI PRISM 310 Genetic Analyzer (Applied Biosystems, USA) using a BigDye Terminator v.3.1 Cycle Sequencing Kit and Big Dye XTerminator Purification Kit (Applied Biosystems). The nucleotide sequences were compared with data stored in GenBank using the basic local alignment search tool (BLAST).

### Calculations and statistical analysis

The results were analysed by Fisher’s exact test using R software v.4.0.2 (R Core Team 2020). The significance threshold was set at α = 0.05. The estimated prevalence of pathogens in pooled samples was calculated using the minimum infection rate (MIR), which assumes that a positive pool contains only a single infected tick (Kahl et al. 1989). The infection rate was also calculated using the maximum likelihood estimation (MLE), using pooledBin from the ‘binGroup’ package (Zhang et al. 2012) with a 95% confidence interval (CI). The MLE calculations are based on the number of the examined pool, the size of pools (number of ticks in a pool), and the number of positive pools.

## Results

### Molecular tick species identification

In total, 1442 ticks were collected, of these 855 were *I. ricinus* (222 females, 204 males, 429 nymphs) and 587 were *D. reticulatus* (350 females, 237 males) (Table S2). A total of 50 isolates of *I. ricinus* and *D. reticulatus* ticks were intended for species confirmation by molecular methods. In all cases, the results of sequencing confirmed morphological identification of the ticks. The sequences of the 16 S rRNA gene fragment obtained in this study showed 100% identity to the sequences from *I. ricinus* and *D. reticulatus* deposited in the GenBank (acc. nos. GU074593.1 and MH636572.1, respectively).

### Prevalence of *Borrelia burgdorferi* s.l.

**Fig. 2:**
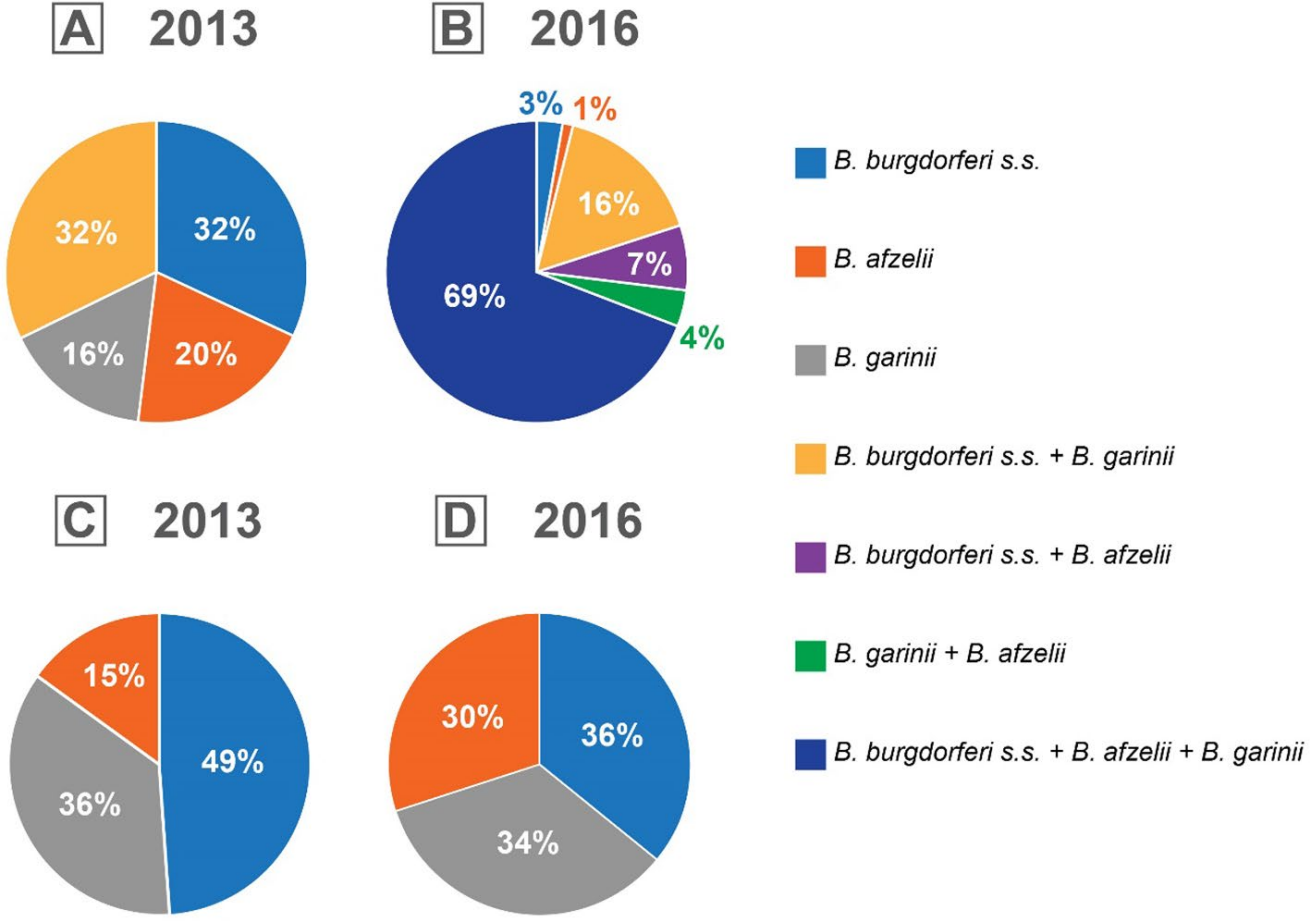
Single and mixed infections (%) of *Borrelia burgdorferi* s.l. species (**A** and **B**), and participation of *B. burgdorferi* s.s., *Borrelia afzelii* and *Borrelia garinii* among the positive results (**C** and **D**) in 2013 and 2016

Comparing the results obtained in 2013 and 2016, the overall percentage of adult ticks infected with *B. burgdorferi* s.l. was at a comparable level (27.9 and 30.4%, respectively). The differences were visible in the distribution of three tested species within the *B. burgdorferi* s.l. complex among each group of adult ticks in particular years. Among *I. ricinus* males and females collected in 2013, infections of single species constituted 68% of *Borrelia* cases with double infections not exceeding 9% in the examined tick population, whereas in 2016, double and triple infections accounted for nearly 96% of all *Borrelia* cases and 29% of all tested groups (Fig. 2). The occurrence of single vs. mixed infections differed significantly (χ^2^ = 59.0, df = 1, *p* < 0.001). The rates of infections among females and males were almost at the same level in the studied years (25.6–30.6%). Taking into consideration both single and mixed infections, both in 2013 and in 2016, the most frequently identified species was *B. burgdorferi* s.s. (17.9 and 28.7%, respectively), followed by *B. garinii* (13.4 and 27.1%) (Table 1). The prevalence of spirochetes in nymphs was lower than in adult ticks. In the case of nymphs examined individually, the percentage of infections was higher (21.6%), compared to the nymphs tested in pools, including both MIR and MLE values (Table 2). The sequences of the *flaB* gene fragments obtained in the presented study was deposited in the GenBank under acc. nos. OL412148 and OL412149 for *B. afzelii*, OL412146 and OL412147 for *B. garinii*, and OL412142–OL412145 for *B. burgdorferi*.

**Table 1:** The occurrence of *Borrelia burgdorferi* s.l. complex species in female and male adult *Ixodes ricinus* ticks (no. positive ticks tested individually, prevalence [%] in parentheses) collected in 2013 and 2016. Ticks collected in 2013 were from Wilków (locality A, see Fig. 1), ticks collected in 2016 were from localities B and C

	2013^a^			2016		
	Females (n = 86)	Males (n = 93)	Total (n = 179)	Females (n = 136)	Males (n = 111)	Total (n = 247)
Single infections and mixed infections						
* B. burgdorferi* s.s.	9 (10.5)	7 (7.5)	16 (8.9)	1 (0.7)	1 (0.9)	2 (0.8)
* B. afzelii*	4 (4.7)	6 (6.5)	10 (5.6)	1 (0.7)	0	1 (0.4)
* B. garinii*	2 (2.3)	6 (6.5)	8 (4.5)	0	0	0
* B. burgdorferi* s.s. + *B. garinii*	7 (8.1)	9 (9.7)	16 (8.9)	6 (4.4)	6 (5.4)	12 (4.9)
* B. burgdorferi* s.s. + *B. afzelii*	0	0	0	4 (2.9)	1 (0.9)	5 (2.0)
* B. garinii* + *B. afzelii*	0	0	0	0	3 (2.7)	3 (1.2)
* B. burgdorferi* s.s. + *B. afzelii* + *B. garinii*	0	0	0	29 (21.3)	23 (20.7)	52 (21.1)
Uninfected ticks	64 (74.4)	65 (69.9)	129 (72.1)	95 (69.9)	77 (69.4)	172 (69.6)
Total ticks infected with *B. burgdorferi* s.l.	22 (25.6)	28 (30.1)	50 (27.9)	41 (30.1)	34 (30.6)	75 (30.4)
Overall occurrence (incl. mixed infections)						
* B. burgdorferi* s.s.	16 (18.6)	16 (17.2)	32 (17.9)	40 (29.4)	31 (27.9)	71 (28.7)
* B. garinii*	9 (10.5)	15 (16.1)	24 (13.4)	35 (25.7)	32 (28.8)	67 (27.1)
* B. afzelii*	4 (4.7)	6 (6.5)	10 (5.6)	34 (25.0)	27 (24.3)	61 (24.7)

^a ^Data previously published by Wójcik-Fatla et al. (2016) (to compare differences in the incidence of single and mixed infections in ticks during the 3 years of the study)

*n* number of examined specimens

**Table 2:** Comparison of minimum infection rate (MIR) and maximum likelihood estimation (MLE) calculated for ticks tested in pools (from localities B and C, see Fig. 1), including 95% confidence intervals (CI) and infection rate in nymphs tested individually (from locality A)

Pathogens	Locality						
	A	B			C		
	%	MIR (%)	MLE		MIR (%)	MLE	
			(%)	95% CI		(%)	95% CI
*Borrelia burgdorferi* s.l.	21.6	15.6	25.4	17.7–35.7	13.0	18.5	11.2–29.2
*B. burgdorferi* s.s.	11.2	13.3	19.4	13.1–27.2	11.3	15.0	8.6–24.3
*B. garinii*	9.7	7.2	8.5	4.8–13.2	12.2	16.7	9.8–26.70
*B. afzelii*	8.2	10.6	13.7	8.7–20.6	8.7	10.5	5.5–12.3
*Neoehrlichia mikurensis*	1.5	0	0	0-2.1	3.5	3.7	1.2–8.7
*B. miyamotoi*	3.0	0.6	0.6	0.03–2.7	2.6	2.7	0.7–7.2
*B. venatorum*	0.7	1.7	1.7	0.5–4.6	0.9	0.9	0.05–4.2

### Prevalence of *Borrelia miyamotoi*

Altogether, 1442 ticks were tested for the presence of *B. miyamotoi*, of which 855 were *I. ricinus* and 587 were *D. reticulatus*. The overall prevalence of *B. miyamotoi* in *I. ricinus* ticks was 2.8% (Table 3). Prevalence of *B. miyamotoi* in nymphs (MIR 1.9%) was almost half that of adult ticks (3.8%), although there was no significant difference in the infection rate between adults and nymphs (χ^2^ = 3.8, df = 2, P > 0.05). There was no difference between the incidence of spirochetes in *I. ricinus* in 2013 and 2016. Analysis of the *p66* and *glpQ* sequences confirmed the presence of *B. miyamotoi* in all positive samples. The sequences of the 438-bp *p66* gene and 329-bp *glpQ* gene determined in this study were deposited in the GenBank under acc. nos. MK977951 and MK977952, respectively. All examined *D. reticulatus* ticks collected from localities C, D, and E were negative for *B. miyamotoi*.

**Table 3:** Occurrence of *Borrelia miyamotoi* and *Neoehrlichia mikurensis* in questing *Ixodes ricinus* ticks collected in 2013 from Wilków (locality A, see Fig. 1) and in 2016 from localities B and C, and *Dermacentor reticulatus* ticks collected in 2010–2012 from localities C, D and E, detected by PCR (no. positive ticks/no. examined ticks (prevalence [%] in parentheses)

Tick’s life stage	*I. ricinus*						*D. reticulatus*	
	2013		2016		2013 + 2016		2010–2012	
	*B. miyamotoi*	*N. mikurensis*	*B. miyamotoi*	*N. mikurensis*	*B. miyamotoi*	*N. mikurensis*	*B. miyamotoi*	*N. mikurensis*
Females	3/86 (3.5)	5/86 (5.8)	7/136 (5.1)	3/136 (2.2)	10/222 (4.5)	8/222 (3.6)	0/350	0/350
Males	2/93 (2.2)	4/93 (4.3)	4/111 (3.6)	6/111 (5.4)	6/204 (2.9)	10/204 (4.9)	0/237	0/237
Nymphs	4/134 (3.0)	2/134 (1.5)	4/295 (1.4^a^)	4/295 (1.4^a^)	8/429 (1.9)	6/429 (1.4)	–	–
Total	9/313 (2.9)	11/313 (3.5)	15/542 (2.8)	13/542 (2.4)	24/855 (2.8)	24/855 (2.8)	0/587	0/587

^a^Results of infection rate in pooled nymphs are presented as minimum infection rate (MIR), i.e., the positive pool was assumed to contain one infected tick

### Prevalence of *Neoehrlichia mikurensis*

*Neoehrlichia mikurensis* DNA was detected in 24 of 855 questing *I. ricinus* ticks with a total prevalence of 2.8% (Table 3). The percentage of infected adult ticks (4.2%) was 3× higher than in nymphs (MIR 1.4%). The prevalence of the pathogen in adults was higher than in nymphs (χ^2^ = 6.9, df = 2, P < 0.05). The sequence of positive samples confirmed the identification of *N. mikurensis*. The sequence of the 407-bp 16 S rRNA gene fragment obtained in the presented study was deposited in the GenBank under acc. nr. MK956058. The DNA of *N. mikurensis* was not detected in any of the 587 examined *D. reticulatus* ticks (collected from localities C, D, E).

### Prevalence of *Babesia* spp.

The presence of *Babesia* was tested in *I. ricinus* ticks collected from localities B and C in 2016 (n = 542). Of the 542 *I. ricinus* ticks from B and C, *Babesia* DNA was detected in 3.0% (Table 4). Infection percentages in adult ticks vs. nymphs did not differ significantly (χ^2^ = 5.8, df = 2, P > 0.05). The sequence of positive samples confirmed the identification of four *Babesia* species. The sequences of *B. microti*, *B. venatorum*, and *B. divergens* were similar to the sequences from ticks obtained in previous studies (Wójck-Fatla et al. 2015). Moreover, one positive sample was 100% identical to *B. capreoli* (GenBank acc. nos. JQ929918 and KP742785). *Babesia microti* prevailed among the identified *Babesia* species (1.5%), followed by *B. venatorum* (1.3%), *B. divergens* (0.2%), and *B. capreoli* (0.2%). The co-occurrence of *B. venatorum* and *B. divergens* was detected in one tick sample.

**Table 4:** Occurrence of *Babesia* species in questing *Ixodes ricinus* ticks collected in 2016 from localities B and C (see Fig. 1) detected by PCR (no. positive ticks, prevalence [%] in parentheses)

Tick’s life stage	*B. microti*	*B. venatorum*	*B. divergens*	*B. capreoli*	Total
Females (n = 136)	4 (2.9)	2 (1.5)	1 (0.7)	1 (0.7)	7^b^ (5.1)
Males (n = 111)	4 (3.6)	1 (0.9)	0	0	5 (4.5)
Nymphs (n = 295)	0	4 (1.4^a^)	0	0	4 (3.6^a^)
Total (n = 542)	8 (1.5)	7 (1.3)	1 (0.2)	1 (0.2)	16 (3.0)

*n* number of examined specimens

^a^Results of infection rate in pooled nymphs are presented as minimum infection rate (MIR), i.e., the positive pool was assumed to contain one infected tick

^b^Co-infection of *B. venatorum* and *B. divergens* in one tick

### Co-infections of pathogens

When assessing the frequency of co-infections in *I. ricinus* ticks, only individually tested adults (n = 426) were taken into account. Due to the fact that some nymphs were isolated in pools, nymphs were excluded from the co-infection analysis. Altogether, at least one pathogen was detected in 159 of 426 adult *I. ricinus* ticks and the co-infections were noticed in 10.1% of all infected ticks. The co-infections with *B. burgdorferi* s.l. and other pathogens were most commonly identified (15 tick specimens). In one tick, the co-occurrence of *B. miyamotoi* and *N. mikurensis* was found. The incidence of co-infections did not differ significantly between 2013 and 2016 (χ^2^ = 1.5, df = 1, P > 0.05) (Table 5).

**Table 5:** Co-infections of *Borrelia burgdorferi* s.l. with other tick-borne pathogens in adult *Ixodes ricinus* ticks (tested individually) collected in 2013 and 2016

Year of collection	Pathogen associations		No. co-infected ticks (%)	
2013 (n = 179)	*B. burgdorferi* s.l.	+ *Neoehrlichia mikurensis*	3 (1.7)	
		+ *B. microti*	1 (0.6)	
		Total	4 (2.2)	
2016 (n = 247)		+ *B. microti*	5 (2.0)	
		+ *B. miyamotoi*	4 (1.6)	
		+ *N. mikurensis*	1 (0.4)	
		+ *B. venatorum* + *B. divergens*	1 (0.4)	
		Total	11 (4.5)	

*n* number of examined specimens

### Estimation of pooled samples

To estimate the prevalence of pathogens in pooled nymphs (from localities B and C), two methods of estimation were applied: MIR and MLE. Assessment of the results showed that in the case of a low prevalence of pathogens in ticks, the results were comparable. Such a situation occurred in the case of results for the *N. mikurensis*, *B. miyamotoi*, and *Babesia* species. In the case of a higher prevalence of a particular pathogen in the examined tick population, MIR and MLE results showed a clear difference, as observed in the example of the Lyme borreliosis spirochetes (Table 2).

## Discussion

The high prevalence of *B. burgdorferi* s.l. in adult *I. ricinus* ticks obtained in this study (ca. 30%) is consistent with the observed tendency of infection rates increasing from western to eastern Europe (Rauter and Hartung 2005). Similar results were presented by Raulf et al. (2018) with almost the same *Borrelia* prevalence in male and female questing ticks (30.6%) based on the meta-analysis studies. A slightly lower percentage obtained for nymphs (MLE ranging from 18.5 to 25.4%), also observed in other studies, could be explained by the fact that nymphs feed on host species much more competent for transmission of *B. burgdorferi* s.l. than adult ticks (Raileanu et al. 2017). Additionally, the relatively high prevalence among the juvenile ticks may be justified by possible transovarial transmission of *Borrelia* spp. among the *I. ricinus* population, reducing the dependence on suitable reservoir hosts (Hauck et al. 2020).

In most studies, species differentiation within the *B. burgdorferi* s.l. complex confirmed that *B. afzelii* followed by *B. garinii* are the most frequent species in Europe (Waindok et al. 2017; Strnad et al. 2017), whereas in the Far East of Russia, the predominance of *B. garinii* over *B. afzelii* has been detected (Pukhovskaya et al. 2019). The most prevalent *B. burgdorferi* s.s. species obtained in the current study is consistent with a previous study from eastern Poland (Wójcik-Fatla et al. 2016); nevertheless, within the last few years an increase in infection rates of *B. afzelii* and *B. garinii* has been noticed. Including single and multiple infections, in 2016, the prevalence of *B. burgdorferi* s.s. and *B. garinii* was almost at the same level (28.7 and 27.1%, respectively) with slightly lower prevalence of *B. afzelii* (24.7%). The predominance of *B. burgdorferi* s.s. species in eastern and southern Poland (Strzelczyk et al. 2015) correlates with studies of Waindok et al. (2017), in which *B. afzelii* occurred most often in ticks removed from human skin, but the questing ticks were more frequently infected with *B. burgdorferi* s.s. However, the domination *B. burgdorferi* s.s. among the *I. ricinus* population in this part of Europe requires further investigation, including diversity and density of the host associated with the *Borrelia* species occurring in particular ecological niches (Cutler et al. 2021). The same conclusion concurs with the very high co-infections rate confirmed in the current study (in 29.5% of *I. ricinus* ticks in 2016), compared with, for instance, 13.6 or 16.7% of mixed infections obtained by Zubriková et al. (2020) and Raileanu et al. (2017), respectively. Taking into consideration that one of the highest and increasing rates of reported human cases of Lyme borreliosis occurs in eastern Poland (infection rate increased from 34.3 to 2010 to 92.0 in 2018 per 100,000 individuals; (National Institute of Public Health-National Institute of Hygiene 2021), common *Borrelia* species co-infections, following Moutailler et al. (2016), seem to be the rule rather than the exception. The incidence of *B. burgdorferi* s.l. in *D. reticulatus* was investigated in a previous study where the prevalence of the pathogen among *D. reticulatus* ticks has not been confirmed (Zając et al. 2017).

In the current study, we found that *B. miyamotoi* and *N. mikurensis* occurred ubiquitously in questing *I. ricinus* ticks from natural areas in eastern Poland. To the best of our knowledge, this is the first report of *B. miyamotoi* and *N. mikurensis* presence in questing *I. ricinus* ticks in this part of the country (Lublin Province). Both bacteria were detected in all three study locations (A, B, C), and all life stages harboured these pathogens. The prevalence of *B. miyamotoi* in *I. ricinus* (2.8%), observed in our study, agrees with previous results from other parts of the country, ranging from 0.3 to 3.9% (Sytykiewicz et al. 2015; Kiewra et al. 2014; Kowalec et al. 2017; Kubiak et al. 2019), as well as from other European countries, such as France (3.0%), the Czech Republic (2.0%), The Netherlands (2.5%), and Switzerland (2.5%) (Cosson et al. 2014; Crowder et al. 2014; Wagemakers et al. 2017; Oechslin et al. 2017). We noticed a slightly higher infection rate in adult ticks than in nymphs, although the difference was not significant. Similarly, previous studies reported no significant differences in prevalence between tick stages. One of the reasons may be both the transstadial and transovarial transmission of *B. miyamotoi*, because it can be transmitted vertically, from infected female tick to offspring (Rollend et al. 2013). In the present study, we found that the causative agents of Lyme borreliosis and *B. miyamotoi* disease occur simultaneously in *I. ricinus* populations; however, the prevalence of *B. miyamotoi* was much lower than *B. burgdorferi* s.l. Similar findings were reported in previous studies in Europe and North America. The differences in infection rates between these two groups of spirochetes may result from various affinities to organs and tissues, disparate duration of the infection in hosts, or detrimental effect of *B. miyamotoi* on survival of ticks (Barbour et al. 2009; Wagemakers et al. 2015). Barbour et al. (2009) reported *B. miyamotoi* infections in 2% of skin biopsies and 6% of blood specimens and *B. burgdorferi* s.l. infections in 76% of skin biopsies and 12% of blood specimens of field-trapped *Peromyscus leucopus* mice. Mice had higher densities of *B. burgdorferi* s.l. in the skin than in the blood, whereas *B. miyamotoi* densities were higher in the blood than in the skin; thus, the acquisition of *B. miyamotoi* by ticks is mostly limited to the period of bacteraemia. Moreover, Taylor et al. (2013) reported that *B. miyamotoi* infection, unlike *B. burgdorferi* species, does not result in persistent infection in rodents. Wagemakers et al. (2016) showed that laboratory mice infected with *B. miyamotoi* produce antibodies against immunogenic proteins of *B. miyamotoi* that eliminate most spirochetes. The reservoir potential of small mammals and the relationship between *B. miyamotoi* and tick vectors and also *B. miyamotoi* and vertebrate hosts need further research.

The overall prevalence of *N. mikurensis* in the current study (2.8%) was similar to results obtained in other European countries, including France (1.7%), the Czech Republic (0.4–4.4%), Slovakia (2.5%), and Austria (4.2%) (Richter and Matuschka 2012; Glatz et al. 2014; Venclíková et al. 2014; Blaňarová et al. 2016). However, in some areas, the prevalence of *N. mikurensis* in ticks collected from vegetation exceeded 20% (Silaghi et al. 2012; Derdáková et al. 2014), and according to Richter and Matuschka (2012), *N. mikurensis* appears to be the second most frequent pathogen transmitted by *I. ricinus* after *B. afzelii* in Europe. In research conducted by Welc-Falęciak et al. (2014), the occurrence of *N. mikurensis* in *I. ricinus* from central and north-eastern parts of Poland (0.2%) was much lower than in the present study. In subsequent years, the percentage of infected individuals (2.9%) collected in similar locations was almost identical to the 2.8% in the current study (Kowalec et al. 2019). We observed a significant difference in infection rate among the life stages, where adult *I. ricinus* ticks were more frequently infected than nymphs. Several other studies reported similar differences in infection rates between adult ticks and nymphs (Jahfari et al. 2012; Silaghi et al. 2012; Venclíková et al. 2014; Kowalec et al. 2019). To date, there is no evidence for transovarial transmission of *N. mikurensis* (Burri et al. 2014), which may be one of the reasons for the higher prevalence range in adults than in nymphs.

In contrast to *I. ricinus* which were infected by *N. mikurensis* and *B. miyamotoi*, none of the 587 *D. reticulatus* ticks harboured those pathogens. Our results correspond to previous studies, in which *D. reticulatus* collected from vegetation were also free from *N. mikurensis* (Richter et al. 2013; Tijsse-Klasen et al. 2013; Hodžić et al. 2017; Szekeres et al. 2015a)d *miyamotoi* infection (Szekeres et al. 2015b; Capligina et al. 2020). To the best of our knowledge, the presence of *N. mikurensis* has been identified in only one specimen of *D. reticulatus* tick collected from vegetation (Krücken et al. 2013). The pathogen’s DNA was also detected in *D. reticulatus* attached to *N. mikurensis*-positive rodents (Silaghi et al. 2012; Obiegala et al. 2014), but this is likely to be the result of the blood meal. This suggests that *D. reticulatus* can acquire the pathogen from an infected animal during the blood meal, but they are incapable of transmitting the pathogen to the subsequent stage transovarially or transstadially. Similarly, the occurrence of *B. miyamotoi* was only detected in one *D. reticulatus* collected from vegetation (Kohn et al. 2019). A low percentage of *D. reticulatus* was also infected with species of the *B. burgdorferi* complex (prevalence ranging from 0 to 3.2%; Nijhof et al. 2007; Richter et al. 2013; Bonnet et al. 2013; Mierzejewska et al. 2015; Kubiak et al. 2016; Zając et al. 2017; Grochowska et al. 2021). Some research indicates that ticks of the genus *Dermacentor* may be unable to maintain and transmit the etiological agents of Lyme borreliosis because of innate immune responses to spirochetes (Johns et al. 2001; Chrudimská et al. 2014). Similar mechanisms may underlie the interaction between *D. reticulatus* and relapsing fever *Borrelia*. The role of *D. reticulatus* in the circulation and maintenance of *N. mikurensis* and *B. miyamotoi* in the environment requires further research. It is important to establish and monitor the vector competence of *D. reticulatus* due to changes of distribution and abundance of this tick species in Poland, where in long-term studies the expansion into new territories and increase in population density have been observed (Buczek et al. 2013; Mierzejewska et al. 2016).

The presence of the *Babesia* species is in agreement with previous findings, where haemoprotozoans were detected in *I. ricinus* and *D. reticulatus* from eastern Poland (Wójcik-Fatla et al. 2012, 2015). Former studies where ticks from locality A were examined have shown a higher incidence of *Babesia* (4.6%) in *I. ricinus* ticks (Wójcik-Fatla et al. 2015). The prevalence rate of *B. microti* in *I. ricinus* prevailed among identified species, as in a previous study (Wójcik-Fatla et al. 2015) and reports from other European countries: Slovakia, Lithuania, and Sweden (Karlsson et al. 2016; Hamšíková et al. 2016; Radzijevskaja et al. 2018). Although most human infections in Europe are attributed to *B. divergens* (Hildebrandt et al. 2013), in the eastern part of the continent, the most common *Babesia* species detected in questing ticks is *B. microti* (Onyiche et al. 2021). The widespread presence of *B. microti* in ticks in this region may result mainly from the common occurrence of their reservoir hosts (mainly rodents) in the environment. The prevalence of *B. microti* infections in small mammals may reach almost 50% (Karbowiak et al. 2004). The prevalence of *Babesia* spp. in *D. reticulatus* collected from localities C, D, and E was examined in a previous study, where the incidence of *Babesia* strains was 2.7%. Two species of *Babesia* were found: *B. microti* and *B. canis* (Wójcik-Fatla et al. 2015).

In the present study, we identified co-infections in 3.8% of adult *I. ricinus* ticks tested individually. The most common co-infection was with the participation of Lyme borreliosis *Borrelia* and another pathogen (15/16). Co-infections in *Ixodes* ticks and their hosts have been reported in North America and Europe, with most cases concerning the coexistence of two out of three pathogenic microorganisms: *B. burgdorferi*, *Anaplasma phagocytophilum*, and *Babesia*. Such double infection affects 1–28% of ticks in Lyme borreliosis endemic areas in the USA, and up to 13% of ticks in Europe (Swanson et al. 2006). The coexistence of pathogens in ticks may pose a risk of acquiring more than one pathogen after a single tick bite. Hence, the characteristic clinical picture of a tick-borne disease caused by one pathogen may be modified by another pathogen (Tokarz et al. 2010). The co-occurrence of borreliosis and babesiosis enhances the severity and prolongs the symptoms of Lyme borreliosis (Diuk-Wasser et al. 2016). The clinical outcome of co-infection of *B. burgdorferi* s.l. with *N. mikurensis* or *B. miyamotoi* remains unknown and needs further investigation.

The limitation of this study was that nymphs from two study locations (B and C) were pooled, whereas nymphs from locality A were examined separately. To assess the level of infection in pooled samples and to reduce analysis error we implemented two methods of estimation MIR and MLE. When comparing the results, it was concluded that in the case of low prevalence the infection rates in both methods were comparable. Thus, for the results of *B. miyamotoi*, *N. mikurensis*, and *Babesia* species, we used MIR as the method of choice. When it comes to the spirochetes of the *B. burgdorferi* s.l. complex, differences in the values of MIR and MLE were considerably greater. Thus, we excluded nymphs from the comparison. Moreover, since the presence of co-infections in pooled ticks may result from the infection of more than one individual, the results referred only to adult ticks tested individually.

## Conclusion

This study indicates that *I. ricinus* ticks are commonly infected with *B. burgdorferi* s.l., *N. mikurensis* and *B. miyamotoi*. Furthermore, lack of infection in questing *D. reticulatus* suggests a negligible role of these ticks in the transmission of these pathogens. The public health significance, particularly in case of *N. mikurensis* and *B. miyamotoi*, may be underestimated due to the unawareness of physicians, an ambiguity of clinical symptoms, and a lack of validated serological diagnostic tests. The coexistence of multiple pathogens in *I. ricinus* may lead to co-infection in humans. Co-infection of Lyme borreliosis with other bacteria or parasites should be considered especially in patients who do not respond properly to antibiotic treatment. The environmental factors and the ongoing climate change have a great impact on the biology and ecology of ticks, and consequently, on tick-borne diseases (Estrada-Peña et al. 2012). Thus, the constant surveillance of ticks and tick-borne pathogens is crucial in assessing the risk of human exposure to infection.

## Supplementary Information

Below is the link to the electronic supplementary material.

Supplementary material 1 (DOCX 18.0 kb)

Supplementary material 2 (DOCX 15.2 kb)
