# Supplementary material for: Prevalence of Borrelia, Neoehrlichia mikurensis and Babesia in ticks collected from vegetation in eastern Poland
Source: Exp Appl Acarol. 2023 Jun 30;90(3-4):409–28. doi: 10.1007/s10493-023-00818-y (PMC10406691; doi:10.1007/s10493-023-00818-y)
Supplement: Supplementary file 1 — Supplementary material 1 (DOCX 18.0 kb) [file 10493_2023_818_MOESM1_ESM.docx]

Table S1 Primers used in the study

| **Target** | **Target gene** | **Fragment size (bp)** | **Outer primer pair (5**ʹ **– 3**ʹ**)** | **Inner primer pair (5**ʹ **– 3**ʹ**)** | **References** |
| --- | --- | --- | --- | --- | --- |
| Tick identification | 16S rRNA | 460 | 16S1: CTGCTCAATGATTTTTTAAATTGCTGTGG |  | Black and Piesman 1994 |
|  |  |  | 16S2: CCATCGATGTGAAYTGCAGGACA |  |  |
| *B.* *microti* | SS-rDNA | 238 | Bab1: CTTAGTATAAGCTTTTATACAGC | Bab2: GTTATAGTTTATTTGATGTTCGTTT | Persing et al. 1992 |
|  |  | 154 | Bab4: ATAGGTCAGAAACTTGAATGATAC | Bab3: AAGCCATGCGATTCGCTAAT |  |
| *Babesia* spp. | 18S rRNA | 422-440 | BabF: GTTTCTGMCCCATCAGCTTGAC |  | Hilpertshauser et al. 2006 |
|  |  |  | BabsR: CAAGACAAAAGTCTGCTTGAAAC |  |  |
| *B.* *divergens* |  | 353 | BabF: GTTTCTGMCCCATCAGCTTGAC |  |  |
|  |  |  | BadR: CAATATTAACACCACGCAAAAATTC |  |  |
| *B.* *venatorum* |  | 362 | BabF: GTTTCTGMCCCATCAGCTTGAC |  |  |
|  |  |  | BeuR: AGACAAGAGTCAATAACTCGATAAC |  |  |
| *B. miyamotoi* | *p66* | 532^*^ | M1: TTCTATATTTGGACACATGTC | M3: CTAAATTATTAAATCCAAAATCG | Geller et al. 2012 |
|  |  |  | M2: CAGATTGTTTAGTTCTAATCCG | M4: GGAAATGAGTACCTACATATG |  |
|  | *glpQ* | 379^*^ | Q1: CACCATTGATCATAGCTCACAG | Q3: GCTAGTGGGTATCTTCCAGAAC |  |
|  |  |  | Q2: CTGTTGGTGCTTCATTCCAGTC | Q4: CTTGTTGTTTATGCCAGAAGGGT |  |
| *B. burgdorferi* s.l. | *flaB* | 482 | FLA1: AGAGCAACTTACAGACGAAATTAAT |  | Wójcik-Fatla ela al. 2016 |
|  |  |  | FLA2: CAAGTCTATTTTGGAAAGCACCTAA |  |  |
| *B. afzelii* |  | 103 | BA1: ATGTTGCAAATCTTTTTG |  |  |
|  |  |  | BA2: TAGCAGGTGTTGGTTGCT |  |  |
| *B. garinii* |  | 125 | BG1: AATCTATTCTCTGGCGAA |  |  |
|  |  |  | BG3: GGAGAATTAACTCCACCC |  |  |
| *B. burgdorferi* s.s. |  | 76 | BB1: AATCTTTTCTCTGGTGAG |  |  |
|  |  |  | BB2: GAGCTCCTTCCTGTTGAA |  |  |
| *N. mikurensis* | 16S rRNA | 488* | EC9: TACCTTGTTACGACTT | IS58-62f: GGAATAGCTGTTAGAAATGACA | Richter and Matuschka 2012 |
|  |  |  | EC12A: TGATCCTGGCTCAGAACGAAC | IS58-594r: CTATCCTCTCTCGATCTCTAGTTT |  |

^*^ The product size was obtained using the inner primers
