# Supplementary material for: Prevalence of Borrelia, Neoehrlichia mikurensis and Babesia in ticks collected from vegetation in eastern Poland
Source: Exp Appl Acarol. 2023 Jun 30;90(3-4):409–28. doi: 10.1007/s10493-023-00818-y (PMC10406691; doi:10.1007/s10493-023-00818-y)
Supplement: Supplementary file 2 — Supplementary material 2 (DOCX 15.2 kb) [file 10493_2023_818_MOESM2_ESM.docx]

**Table S2** Sample collection data

|  | ***Ixodes ricinus*** | | | | | | |
| --- | --- | --- | --- | --- | --- | --- | --- |
|  |  |  |  | Tick’s life stage | | |  |
| Locality | GPS data | Habitat | Collection year | Females | Males | Nymphs | Total |
|  |  |  |  | Number of collected ticks | | | |
| **A** | 51º 15' 33.341" N 21º 52' 12.759" E | Deciduous forest, meadows and idle land covered with bushes and trees | 2013 | 86 | 93 | 134 | 313 |
| **B** | 51º 5' 21.695" N 22º 28' 34.073" E | Mixed deciduous forest with rich undergrowth and with the adjacent meadows | 2016 | 41 | 38 | 180 | 259 |
| **C** | 51º 29' 53.277" N 23º 24' 34.413" E | Coniferous and mixed forests with poor undergrowth with adjacent meadows | 2016 | 95 | 73 | 115 | 283 |
|  |  | **Total** | | **222** | **204** | **429** | **855** |
|  | ***Dermacentor reticulatus*** | | | | | | |
| **C** | 51º 29' 53.277" N 23º 24' 34.413" E | Coniferous and mixed forests with poor undergrowth with adjacent meadows | 2012 | 122 | 35 | - | 157 |
| **D** | 51º 38' 20.4" N 22º 53' 60" E | Meadows, wastelands, idle lands covered with trees and bushes, the edges of a mixed forest | 2010 | 147 | 135 | - | 282 |
| **E** | 51º 29' 49.631" N 22º 51' 19.686" E | Meadows, pastures and idle lands covered with trees and bushes | 2011 | 81 | 67 | - | 148 |
|  |  | **Total** | | **350** | **237** | **-** | **587** |
